# Comparison of Canine and Feline Adipose-Derived Mesenchymal Stem Cells/Medicinal Signaling Cells With Regard to Cell Surface Marker Expression, Viability, Proliferation, and Differentiation Potential

**DOI:** 10.3389/fvets.2020.610240

**Published:** 2021-01-13

**Authors:** Metka Voga, Valerija Kovač, Gregor Majdic

**Affiliations:** ^1^Veterinary Faculty, Institute for Preclinical Sciences, University of Ljubljana, Ljubljana, Slovenia; ^2^Blood Transfusion Centre of Slovenia, Ljubljana, Slovenia; ^3^Medical Faculty, Institute for Physiology, University of Maribor, Maribor, Slovenia

**Keywords:** mesenchymal stem cells, dog, cat, comparison, proliferation, differentiation, cell surface marker, viability

## Abstract

Remarkable immunomodulatory abilities of mesenchymal stem cells, also called multipotent mesenchymal stromal cells or medicinal signaling cells (MSCs), have entailed significant advances in veterinary regenerative medicine in recent years. Despite positive outcomes from MSC therapies in various diseases in dogs and cats, differences in MSC characteristics between small animal veterinary patients are not well-known. We performed a comparative study of cells' surface marker expression, viability, proliferation, and differentiation capacity of adipose-derived MSCs (ADMSCs) from dogs and domestic cats. The same growth media and methods were used to isolate, characterize, and culture canine and feline ADMSCs. Adipose tissue was collected from 11 dogs and 8 cats of both sexes. The expression of surface markers CD44, CD90, and CD34 was detected by flow cytometry. Viability at passage 3 was measured with the hemocytometer and compared to the viability measured by flow cytometry after 1 day of handling. The proliferation potential of MSCs was measured by calculating cell doubling and cell doubling time from second to eighth passage. Differentiation potential was determined at early and late passages by inducing cells toward adipogenic, osteogenic, and chondrogenic differentiation using commercial media. Our study shows that the percentage of CD44^+^CD90^+^ and CD34^−/−^ cells is higher in cells from dogs than in cells from cats. The viability of cells measured by two different methods at passage 3 differed between the species, and finally, canine ADMSCs possess greater proliferation and differentiation potential in comparison to the feline ADMSCs.

## Introduction

In recent years, significant interest for stem cell therapies has arisen in veterinary medicine. It has become evident that mesenchymal stromal cells' or medicinal signaling cells' (MSCs') therapeutic actions are the result of their complex immunomodulatory abilities, including paracrine action (1), secretion of extracellular vesicles (2–4), apoptosis mediated immunomodulation (5), and mitochondrial transfer of membrane vesicles and organelles (6, 7). In dogs and cats, most common veterinary patients, notable positive outcomes have been reported from MSC therapies of various diseases such as orthopedic diseases (8–12), feline chronic gingivostomatitis (13–15), inflammatory bowel disease (16, 17), and skin diseases (18). MSCs have been isolated from various tissues such as adipose tissue (19–23), bone marrow (20, 21, 23–25), synovium (20), synovial fluid (19, 26), umbilical cord (27), Wharton's jelly (28), peripheral blood (29), muscle, and periosteum (30). Adipose tissue is generally considered the most attractive source of MSCs because of a large MSC yield and minimally invasive procedure needed for cell harvesting (31). After their isolation, MSCs are characterized to prove their mesenchymal nature. In addition to plastic adherence, minimal criteria to define human MSCs, set by the Mesenchymal and Tissue Stem Cell Committee of the International Society for Cellular Therapy, include their ability to differentiate into osteoblasts, adipocytes, and chondroblasts. Also, MSCs must express CD105, CD73, and CD90 and lack the expression of CD45, CD34, CD14 or CD11b, CD79a or CD19, and HLA class II (32) surface markers.

While all animal MSCs are plastic-adherent and show trilineage differentiation potential, not all express the same panel of surface antigens. Previous MSC studies showed that canine (19, 33–35) and feline MSCs (22, 36) consistently express CD44 CD90 and lack CD34 expression, while the expression of other markers varies. Standards to define animal MSCs are therefore not yet established. Clinical administration of MSCs usually entails expanding cells *in vitro* to obtain a sufficient number of cells. It is well-known that MSC populations are intrinsically heterogeneous what can significantly impact their therapeutic potency (37). Besides different factors, such as MSC source (19, 21, 23, 38), tissue collection site (39–41), animal age (39, 42–44), and the number of passages (45–48) that have been demonstrated to affect MSC characteristics *ex vivo*, animal species could likely also influence MSC potency. Only a few studies compared MSCs between species directly (49–51), while it is difficult to draw any comparisons between different studies using cells from different species because of the lack of standardization of methods for the isolation, culture, and characterization of animal MSCs. As differences in stem cell properties between different animal species might lead to the differences in the stem cell therapy's success, they should be therefore explored. In addition to the importance of studying interspecies differences, investigating sex differences is another critical aspect of scientific research, although it is often neglected in preclinical studies (52). It is well-established that the sex of a patient can affect the risk for both disease susceptibility and progression (53). Sex differences have also been found in stem cell biology and therapeutic efficacy in different species (54–57). Studying both males and females is therefore necessary in different studies including cells, animals, and humans, as it may lead to novel targets for disease modifiers (53). The aim of our study was to determine differences in adipose-derived MSCs (ADMSCs) properties from dogs and cats of both sexes in terms of their surface marker expression, viability, and proliferation and differentiation capacity. Although no sex differences were found between the species in the study, the results of our study show important differences in MSC proliferation and differentiation potential between dogs and cats. The results of our study show that species as a factor must be considered when planning the preparation of canine and feline ADMSCs for clinical applications.

## Materials and Methods

### Animals and Adipose Tissue Collection

Adipose tissue was individually collected from 11 dogs (aged 6 months to 2 years, four males and seven females) and eight cats (aged 3 months to 1 year, four males and four females) during routine castration or ovariohysterectomy at the Small Animal Clinic of the Faculty of Veterinary medicine in Ljubljana and from the Veterinary Clinic of Biotechnology educational center in Ljubljana. All animals were healthy, with no known comorbidities. In all animals, adipose tissue was obtained from the abdominal subcutaneous region. All animals were client-owned, and all owners agreed to the collection of tissue and signed informed consent. As the study was conducted on client-owned animals undergoing a routine clinical procedure with the owner's approval to collect a small piece of adipose tissue, no other approval of the ethical committee was needed according to Slovenian legislation and official opinion from the Administration of the Republic of Slovenia for Food Safety, Veterinary and Plant protection responsible for issuing ethical permits for animal experiments.

### Isolation of ADMSCs

Adipose tissue was washed with Dulbecco phosphate-buffered saline (DPBS, Gibco, USA) and cut with a scalpel into small pieces. Adipose tissue was then incubated overnight at 37°C in Dulbecco modified eagle medium (DMEM, Gibco, USA) containing 0.1% collagenase type II (Sigma–Aldrich, DE). The digested tissue was centrifuged at 240 g for 4 min, and the supernatant was discarded. The pellet of cells was resuspended in a cell culture medium containing DMEM and 10% fetal bovine serum (Gibco, USA). The cell suspension was plated into six-well plates (TPP, Switzerland) as passage 0 (P0) and cultured at 37°C in a 5% CO_2_ incubator. The cell culture medium was changed every 2–3 days. After 70–90% confluency was reached, cells were trypsinized and further processed for flow cytometry, proliferation, and differentiation assays.

### Flow Cytometry (Fluorescence-Activated Cell Sorting) Analysis

For fluorescence-activated cell sorting (FACS) analysis, 1 × 10^6^ cells from passage 3 were used to detect the expression of cell surface markers CD44, CD90, and CD34. Following trypsinization, cells were counted, centrifuged (240 g for 4 min), and washed twice with DPBS. Cells were kept in suspension in DPBS at 4°C overnight. The following day, cells were transferred to the Blood Transfusion Center of Slovenia. Cells were stained with the following antibodies: APC conjugated against CD44 (antibody clone IM7, 103012, Biolegend, USA) and fluorescein isothiocyanate conjugated against CD34 (antibody clone 581, 60013FI, Stemcell Technologies, USA) for both canine and feline ADMSCs, PE conjugated against CD90 (antibody clone YKIX337.217, 12-5900-42, eBioscience, USA) for canine ADMSCs, and PE conjugated against CD90 (antibody clone 5E10, 555596, BD bioscience, USA) for feline ADMSCs. For antibody titration, 1, 2, 3, 4, 5, and 10 μL of each antiserum per 100 μL of 1 × 10^6^ cells were used. Appropriate dilutions of antibodies used for staining are shown in Table 1. Cells were then vortexed, incubated at room temperature in the dark for 10 min, washed twice with DPBS, vortexed, and centrifuged again (500 g for 5 min). The supernatant was decanted. Finally, cells were resuspended in 300 μL of DPBS for FACS analysis. The exclusion of non-viable cells was performed by staining cells with a 7-amino-actinomycin D solution (Miltenyi Biotec, USA). Experimental settings were set up using unstained cells, single color stain, and Fluorescence Minus One controls. A minimum of 20,000 events was recorded. Cells were analyzed with a BD FACSAria flow cytometer (BD Bioscience). FACSDiva 8.0.1 software (BD Bioscience) was used for FACS data analysis.

**Table 1:** Data on antibodies and their dilutions used for FACS analysis in the study.

**Cell surface marker**	**Conjugation**	**Antibody clone**	**Isotype**	**Target species**	**Catalog number**	**Source**	**Antibody dilution (canine ADMSCs)**	**Antibody dilution (feline ADMSCs)**
CD34	FITC	581	Mouse IgG1	Human	60013FI	Stemcell Technologies, USA	1: 20	1: 20
CD44	APC	IM7	Rat IgG2b	Mouse, human	103012	Biolegend, USA	1: 66	1: 400
CD90	PE	YKIX337.217	Rat IgG2b	Dog	12-5900-42	eBioscience, USA	1: 20	/
CD90	PE	5E10	Mouse IgG1	Human	555596	BD bioscience, USA	/	1: 66
CD90	PE	5E10	Mouse IgG1	Human	60045PE	Stemcell technologies, USA	/	/
CD105	PE-Cy7	SN6	Mouse IgG1	Human	25-1057-42	eBioscience, USA	/	/

*CD, cluster of differentiation; FITC, fluorescein isothiocyanate; PE, phycoerythrin; APC, allophycoerythrin; PE-CY7, phycoerythrin–cyanine 7; Slash (/), non-reactive*.

### Proliferation Potential Assay

After 70–90% confluency was reached in each passage, cells were trypsinized to the single cell and seeded at the density of 10e^4^ cells per cm^2^ as the next passage into a new T25 cell culture flask (TPP). Cells in the cell culture were maintained up to the eighth passage (P2–P8). At each passage from second to eighth, the number of cells at seeding and harvesting was determined with a hemocytometer, and cell viability was assessed using trypan blue dye. Cell doubling (CD) and cell doubling time (CDT) were calculated using the following formulas:

*CD* = *log (Nt/N0)* × *3.32*

*CDT* = *T/CD*

*C(CD)* = *CD(P1)*+*CD(P2)*+....+*CD(P10)*

where *Nt* is the number of cells at harvesting, *N0* is the number of cells at seeding, *T* is the time of cell culture for each passage, *CD* is the number of cells' doublings at one passage, *C(CD)* is the cumulative CD of all passages, and CDT is the time needed for a cell number to double (58).

### Cell Viability

Cell viability was measured by two methods. During proliferation potential assay, viability was measured with hemocytometer immediately after cell trypsinization using trypan blue dye, at each passage from second to eighth. At passage 3, viability was also measured during FACS analysis using 7-amino-actinomycin D solution to exclude non-viable cells from the surface marker expression analysis and to compare the effect of additional manipulation and overnight storage on cells from both species.

### Differentiation Potential Assay

Differentiation potential was assessed by inducing cells into adipocytes, osteocytes, and chondrocytes. Differentiation potential was assessed at early (P2) and late (P8 for canine ADMSCs and P6 for feline ADMSCs) passages. For the adipogenic differentiation, 4 × 10^4^ cells were seeded in 12-well plates. The day after seeding, the cell culture medium was removed. Adipogenic (StemPro Adipogenesis Differentiation Kit, Gibco, USA) medium was added and changed every 2–3 days. The cell culture medium was added to the wells that served as negative controls. Adipogenic differentiation was analyzed with oil-red-O staining (Sigma–Aldrich, DE) after 14 days of culturing, following standard procedure. For the osteogenic differentiation, 4 × 10^4^ cells were seeded in 12-well plates. After 90–100% confluency was reached, the cell culture medium was removed. Osteogenic (StemPro Osteogenesis Differentiation Kit, Gibco, USA) medium was added and changed every 2–3 days. Osteogenic differentiation was analyzed with alizarin red S staining (Sigma–Aldrich, DE) following standard procedure after 14 days of culturing. For the chondrogenic differentiation, micromass cultures were generated by seeding 5-μL droplets of 4 × 10^4^ cells in the center wells of the 12-well plate. After cultivating micromass cultures for 6 h under high humidity conditions, a chondrogenic medium (StemPro Chondrogenesis Differentiation Kit, Gibco, USA) was added to culture vessels. The cell culture medium was added to the wells that served as negative controls. Micromass cultures were incubated at 37°C in an incubator with 5% CO_2_ and a humid atmosphere. The medium was changed every 2–3 days. Chondrogenic differentiation was analyzed with Alcian blue staining (Sigma–Aldrich, DE) following standard procedure after 14 days of culturing. Differentiated cells were then visualized under light microscope.

### Light Microscopy and Analysis

For analysis of multilineage differentiation potential of canine and feline ADMSCs, an inverted microscope (Nikon Eclipse TS100, Nikon, Japan) with Nikon Digital Sight DS-U2 camera was used. Images were captured in NIS-Elements D3.2 Live quality program. Images of adipogenic differentiation were captured at 400× magnification and qualitatively analyzed. Images of osteogenic and chondrogenic differentiation were captured at 40× magnification. Seven view fields in one well of a 12-well plate were randomly selected and quantitatively analyzed by measuring the area of differentiated cells. In the ImageJ program (59), images were converted to binary type and then segmented using DynamicThreshold_1d.class plugin (60), displaying (max + min)/2 images. The area of particles larger than 100 μm^2^ was measured in each field view, and the total area covered by differentiated cells was calculated.

### Statistical Analysis

All statistical analyses were performed with the NCSS software package (Kaysville, UT, USA). The normality of the data was checked by the Kolmogorov–Smirnov test for normality. One-way analysis of variance (ANOVA) was used to determine differences in the cell surface marker expression and the viability in the third passage with species and sex as independent variables. Proliferation capacity and viability throughout the experiment were analyzed by repeated-measures ANOVA with species and sex as independent variables and passage as within factor. Differences in differentiation were determined by repeated- measures ANOVA with sex and species as independent variable and optic field as within factor. Differences in differentiation were determined separately for early and late passages. Additionally, the *post-hoc* Tukey–Kramer multiple-comparisons test was used to clarify the differences between particular pairs further. Statistical significance was determined with *p* < 0.05.

## Results

### Cell Culturing and Proliferation Potential of MSCs

**Figure 1:**
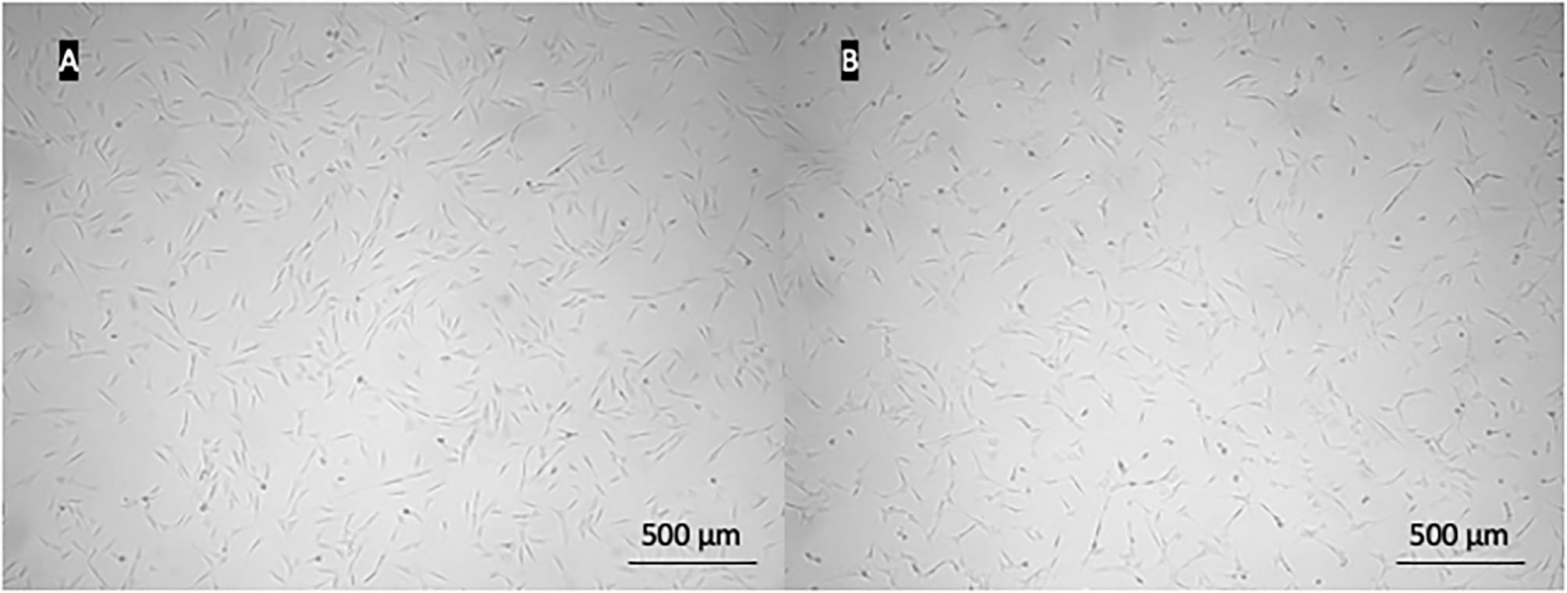
Images of canine **(A)** and feline **(B)** ADMSCs. Cells from both species exhibited similar spindle-shaped fibroblast-like morphology.

**Figure 2:**
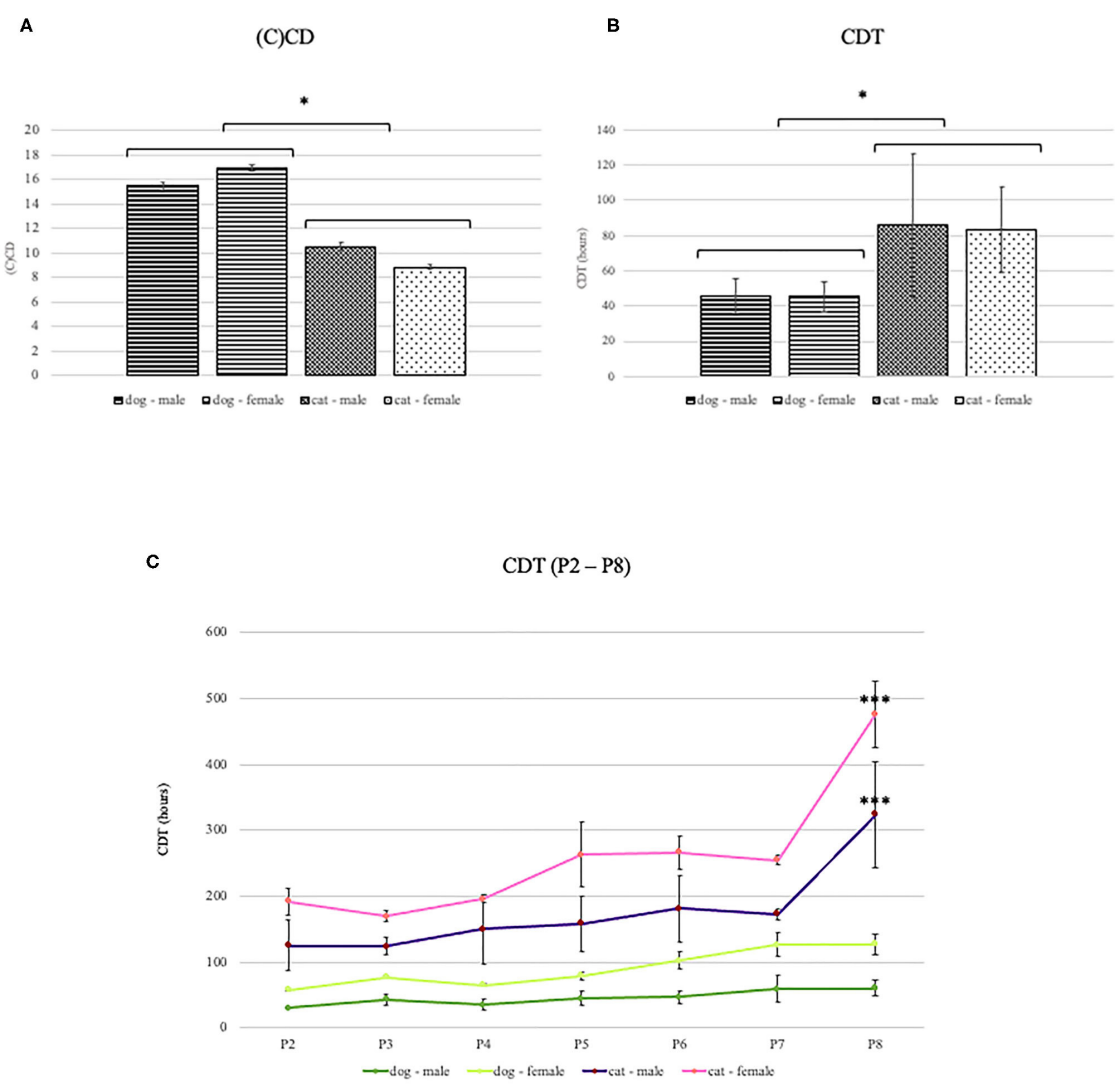
(C)CD and CDT of canine and feline ADMSCs. Canine ADMSCs (C)CD was statistically significantly higher than feline ADMSCs (C)CD (**A**, *p < 0.01), and average CDT was statistically significantly lower for canine than for feline ADMSCs (**B**, *p < 0.05). No sex differences in CD or CDT were observed in either of the species. Interestingly, CDT in canine ADMSCs increased gradually through passages, whereas CDT in feline ADMSCs increased unevenly. In dogs, no statistically significant differences were observed between passages. Interestingly, CDT increased significantly in passage 8 in cats only and was different from all other feline and canine passages (***p < 0.001; **C**). Results are presented as mean ± SEM.

The adipose tissue was successfully collected from all animals. Under the light microscope, cells from both species appeared spindle-shaped with the fibroblast-like morphology (Figure 1). Cells were maintained up to the eighth passage. We attempted to grow cells for longer, but after the eighth passage, most cells stopped proliferating or proliferated very slowly. Therefore, all analyses were performed with cells up to the eighth passage. At each passage from second to eighth, CD and CDT were determined. Cumulative CD [(C)CD)] of canine ADMSCs was significantly higher than (C)CD of feline ADMSCs (*p* < 0.01; Figure 2A), and average CDT was significantly shorter for canine ADMSCs than for feline ADMSCs (*p* < 0.05; Figure 2B). An increase in CDT from the second to the eighth passage was relatively gradual in canine ADMSCs in comparison to CDT of feline ADMSCs, which increased unevenly. Interestingly, CDT increased significantly in passage eight in cats only and was different from all other feline and canine passages (*p* < 0.001; Figure 2C). There were no statistically significant differences in CD or CDT between the sexes of both species.

### Flow Cytometry Analysis

Undifferentiated ADMSCs at passage 3 were evaluated for the expression of cell surface markers CD44, CD90, and CD34 (Table 1). We also tested antibodies against CD105 (Table 1); however, these antibodies did not work with our cells and were therefore not included in the analyses.

**Figure 3:**
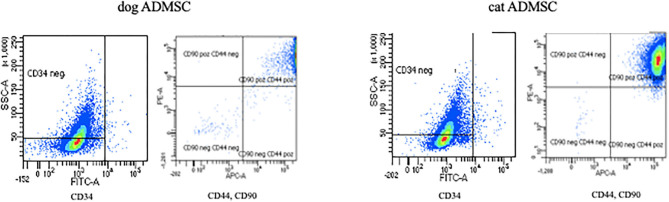
Expression of canine and feline ADMSC surface markers analyzed by FACS at passage 3. Both canine and feline ADMSCs expressed CD44 and CD90 but did not express CD34.

**Figure 4:**
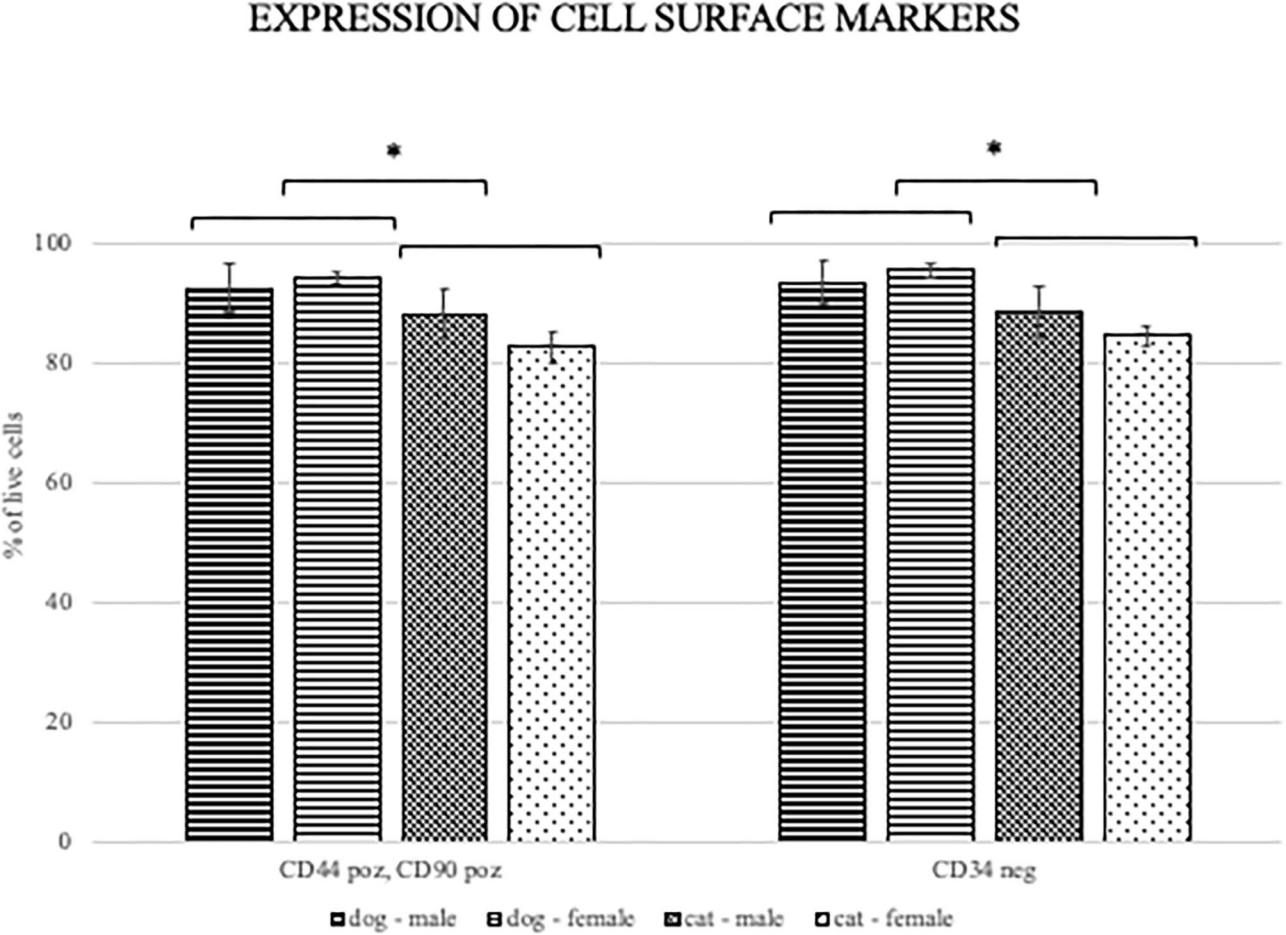
Percentage of CD440^+^CD90^+^ and CD34^−/−^ canine and feline cells. Percentage of live ADMSCs expressing CD44 and CD90 was statistically significantly higher in canine than in feline cells (*p < 0.01). The percentage of live CD34 negative ADMSCs was also statistically significantly higher in canine than in feline cells (*p < 0.05). There were no differences in cell marker expression between the sexes of ADMSCs from either of the species. Results are presented as mean ± SEM.

FACS analysis revealed that the most canine and feline ADMSCs were positive for CD44 and CD90 and negative for CD34 (Figure 3). Percentage of live CD44^+^CD90^+^ ADMSCs was statistically significantly higher in canine than in feline cells (*p* < 0.01). The percentage of live CD34^−/−^ ADMSCs was also statistically significantly higher in canine than in feline cells (*p* < 0.05; Figure 4). No differences in cell marker expression between sexes were observed in either of the species.

### Cell Viability

**Figure 5:**
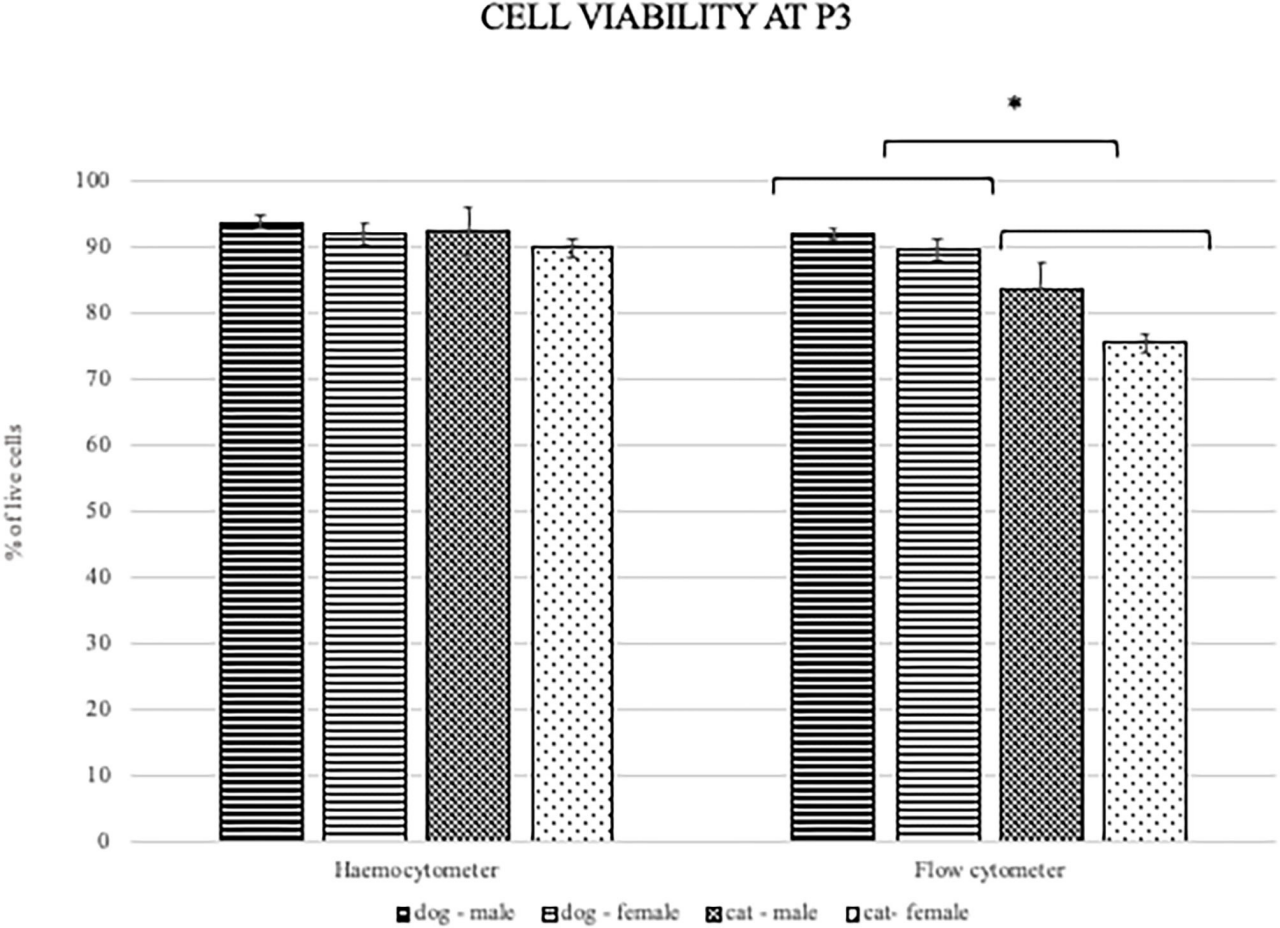
Viability of canine and feline ADMSCs measured by hemocytometer (left) and flow cytometry (right) at passage 3. When measured by hemocytometer, the viability of ADMSCs was similar between both species. When measured by flow cytometry, the viability of feline ADMSCs was statistically significantly lower than that of canine ADMSCs (*p < 0.01). No differences in cell viability between sexes from either species were present, regardless of the method. Results are presented as mean ± SEM.

MSC viability was determined with the hemocytometer using trypan blue at each passage during the proliferation potential assay. Additionally, viability was determined at passage 3 with flow cytometry using the 7-amino-actinomycin D staining solution to exclude non-viable cells from the surface marker expression analysis and to compare the effect of additional manipulation and overnight storage on the cells from both species. Mean viability of ADMSCs measured with the hemocytometer during proliferation ranged from 93 to 96% in the second passage and from 82 to 88% in the eighth passage (Table 2), with no statistically significant difference between canine and feline cells, although there was a trend toward statistical significance with better viability of canine ADMSCs. Also, no differences in viability between the sexes of both species were observed. Contrary to the viability measured during proliferation assay, flow cytometry results showed a statistically significant difference in the viability between canine and feline ADMSCs in passage 3, with cells from dogs showing higher viability than cells from cats (*p* < 0.01). The mean viability of canine and feline ADMSCs in passage 3 was 90.44 and 79.67%, respectively (Figure 5).

**Table 2:** Average viability (%) of canine and feline ADMSCs measured by hemocytometer during proliferation potential assay at each passage (P2–P8).

**Passage**	**P2**	**P3**	**P4**	**P5**	**P6**	**P7**	**P8**
Dog cells	96	93	94	92	91	86	88
Cat cells	93	91	91	92	89	76	82

### Differentiation Potential of MSCs

Canine and feline ADMSCs were induced toward trilineage differentiation at early (P2) and late (P8 for canine and P6 for feline ADMSCs) passage. Late passage for feline ADMSCs was determined as passage 6, as these cells were able to differentiate at passage 6 but not at later passages. Although late passages are different and therefore not directly comparable, our aim was to determine differentiation capacity toward the end of the proliferative capacity of ADMSCs, and as these were different between dogs and cats, we chose different passages for late differentiation.

**Figure 6:**
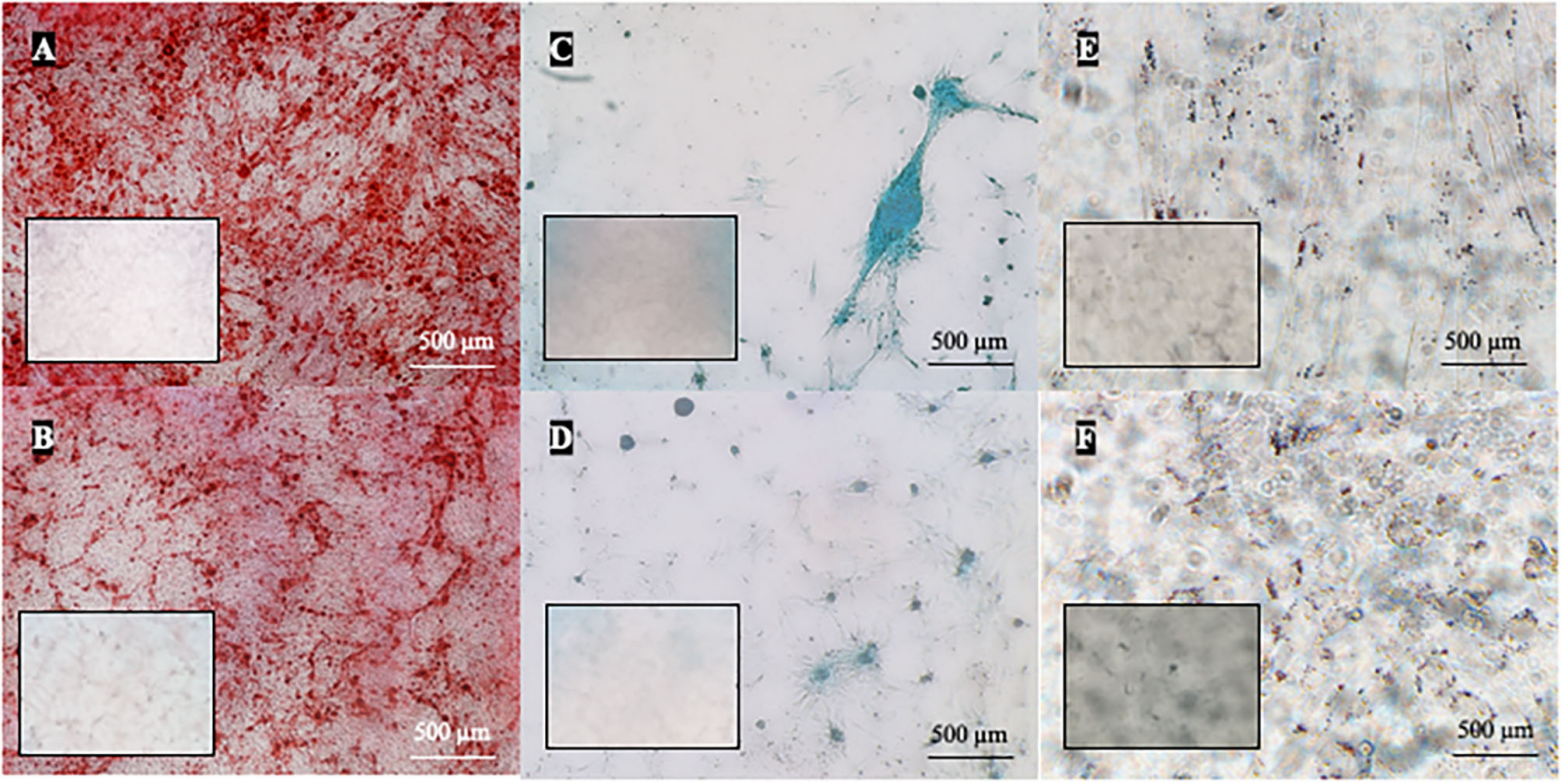
Multilineage differentiation of cells in passage 2. ADMSCs from cats and dogs successfully underwent osteogenic **(A,B)**, chondrogenic **(C,D)**, and adipogenic differentiation **(E,F)**. In osteogenic differentiation, mineral deposits in the extracellular matrix were stained red by alizarin-red-S (dog **A**, cat **B**). Chondrogenic differentiation is indicated by the formation of chondrogenic nodules that stain blue with Alcian blue (dog **C**, cat **D**). Red intracellular lipid droplets stained with oil-red-O are indicative of adipogenic differentiation (dog **E**, cat **F**). Respective negative controls are shown as inserts in each photomicrograph.

**Figure 7:**
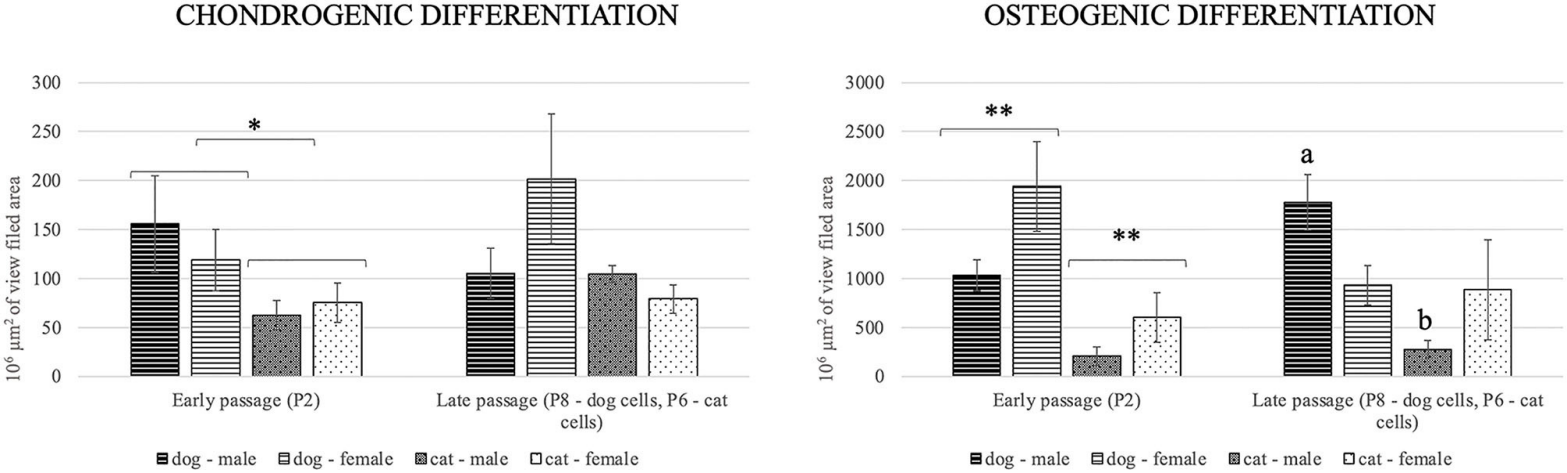
Chondrogenic and osteogenic differentiation at early (P2) and late (P8 for canine cells and P6 for feline cells) passages. Positively stained area in chondrogenic differentiation was statistically significantly more extensive in canine ADMSCs than in feline ADMSCs at passage 2 (*p < 0.05). In osteogenic differentiation, there was a statistically significant difference between the sexes of both species (**p < 0.01) but not between species at passage 2, although there was a statistical trend for difference between species (*p* = 0.07). In late passages, there was statistically significant difference in osteogenic differentiation between the species, but only in males (a different from b, *p* < 0.01).

Osteogenic differentiation led to mineral deposits in the extracellular matrix staining red with alizarin-red-S (Figures 6A,B). After chondrogenic differentiation, proteoglycans in the extracellular matrix of layered cell clusters stained positive with Alcian blue (Figures 6C,D). Adipogenic differentiation resulted in the formation of intracellular lipid droplets staining red with oil-red-O (Figures 6E,F). Both canine and feline ADMSCs differentiated into adipocytes, osteocytes, and chondrocytes at early (P2) and late passages (P8 for canine cells and P6 for feline cells). Adipogenic differentiation was assessed qualitatively as quantification of adipogenic differentiation was thwarted because of the very small size of lipid droplets that needed to be visualized under the large magnification. No apparent differences in the adipogenic differentiation based on the qualitative assessment between canine and feline cells were observed. Osteogenic and chondrogenic differentiated cells were analyzed with the ImageJ program wherein the area of particles larger than 100 μm^2^ was measured and compared between canine and feline ADMSCs. Results showed that the total positively stained area in chondrogenic differentiation was statistically significantly more extensive in canine ADMSCs than in feline ADMSCs at passage 2 (early passage; *p* < 0.05, Figure 7), and there was a statistical trend for difference between species in osteogenic differentiation at passage 2 (early passage; *p* = 0.07). Interestingly, there was a statistically significant difference between sexes in osteogenic potential in passage 2 (early passage) with cells from female dogs and cats showing larger osteogenic potential than cells from males of both species (*p* < 0.01, Figure 7). In late passages, we found statistically significant difference in the osteogenic potential between species, although curiously *post-hoc* test revealed that this difference was only present in males with male dogs showing higher osteogenic potential than male cats (*p* < 0.01, Figure 7).

## Discussion

Despite substantial progress in veterinary regenerative therapy in recent years, understanding MSC behavior and their mechanisms of action is an ongoing process. MSCs differ in various characteristics with regard to the tissue source (19, 21, 23, 38), anatomical location (39–41), animal age (39, 42–44), and their characteristics change with the number of passages (45–48). There are very limited data about differences between MSCs from different species. However, one mode of cell therapy used in certain species could not be necessarily directly applicable to another species. In the present study, we examined characteristics of adipose-derived MSCs from two species, both common veterinary patients, cats and dogs.

We performed a comparative study of surface marker expression, viability, proliferation, and differentiation capacity between canine and feline ADMSCs, with the same media and methods used for the isolation, cell culture, and characterization of ADMSCs of both species. Both canine and feline ADMSCs exhibited similar spindle-shaped fibroblasts–like morphology. Although animal MSCs are plastic adherent and able to differentiate not only into adipocytes, chondrocytes, and osteocytes but also into other lineages such as neuronal lineage (61, 62), there are no minimal criteria set to define animal MSCs based on the surface antigens as are for human MSCs (32). Previous MSC studies mostly described ADMSCs from dogs (19, 33–35) and cats (22, 36) as consistently CD34-negative and -positive for CD44 and CD90. Expression of some other markers, essential to define human MSCs, varies in animal MSCs. The expression of CD105 and CD73 in canine and feline MSCs varies depending on the tissue of origin (19) or is expressed only in dogs (38, 63) but not in cats (36).

The aim of our study was to compare the expression of surface markers that are known to be consistently expressed on MSCs from both species. Therefore, we used markers CD34, CD44, and CD90. We also considered two other markers (CD105 and CD73) that define human MSCs (32), but were not included in the final study. Marker CD105 was not used as we were unable to find commercially available anti-canine or anti-feline CD105 antisera. However, we tested antibodies against CD105 that were reportedly used in one previous study (64), but we could not obtain positive signal despite extensive optimization with our canine or feline cells. Marker CD73 was not included in the study as it was shown before that it is not expressed in feline cells and was therefore not considered as a marker consistently expressed in both species. However, as there are no reports on the comparisons of relative marker expression between species, future studies should also consider more thorough investigations of MSC surface marker expression differences between various animals, especially when species-specific MSC markers will hopefully become available in the future.

In line with the results of previous studies, we showed that most of the canine and feline ADMSCs express MSC surface markers CD44 and CD90 and lack the expression of hematopoietic marker CD34. Interestingly, the percentage of cells expressing CD44 and CD90 and not expressing CD34 was statistically significantly higher in canine ADMSCs than in feline ADMSCs. It was previously described that MSCs exhibit donor-to-donor and intrapopulation heterogeneity and that MSC populations consist of distinct subpopulations, so the properties of MSC populations cannot be ascribed to single cells (37). One possible reason for the difference in relative surface marker expression between canine and feline cells in our study might be that feline cell populations are more intrinsically heterogeneous than canine cells.

We measured the viability of cells by two different methods, hemocytometer during proliferation potential assay immediately after trypsinization at each passage and by flow cytometer during surface marker expression analysis at passage 3 after 1 day of handling. The cell viability measured by two methods was used to compare the effect of additional manipulation and overnight storage on the cells from both species. The viability of canine ADMSCs in passage 3, measured by flow cytometry, was statistically significantly higher than the viability of feline ADMSCs. However, the viability of both canine and feline cells, measured with trypan blue through the passages, was not significantly different at any passage. Several studies showed that viability determined by microscopy and flow cytometry contributes to similar results and that correlation data are in good agreement with both methods (65–67). Therefore, it is unlikely that this difference was caused by the difference in methods, but rather by differences in handling the cells. While cells for trypan blue staining were counted almost immediately after trypsinization, cells for flow cytometry underwent much longer manipulation, including overnight storage at 4°C in DPBS suspension and additional manipulation of cells prior to flow cytometry analysis. Although these differences will have to be confirmed in further studies, the results suggest that feline cells might be more sensitive to handling and storage in suboptimal conditions. This could have important implications for potential clinical use of MSCs in veterinary medicine, as any such use inadvertently involves the transport of live cells. If feline cells are more sensitive to handling and storage, this will have to be considered.

The life span of MSCs cultured *ex vivo* is limited, and it has been demonstrated in several studies that serial passages alter their multipotent properties (45–48). The ability of MSCs to self-renew is thus an important feature to be analyzed *in vitro* and can be done by calculating CD and CDT—number of cells' doublings in one passage and time needed for a cell number to double. It has been shown in several studies that CD decreases and CDT increases with passages in cells from various species (36, 38, 45, 68). Similarly, the results of our study showed that the proliferation capacity of both canine and feline ADMSCs decreased with passages. Interestingly, the proliferation capacity of canine ADMSCs seems to decrease very gradually with passages, whereas the proliferation capacity of feline ADMSCs was much more varied between passages. This variation between passages was observed in all feline samples and therefore suggests a real effect, although it is difficult to explain what might cause such non-linear difference between passages.

In previous studies, proliferation potential was shown to depend on various factors, including tissue of origin and anatomical site of the tissue collection. There might also be a difference between species regarding MSC proliferation and differentiation potential, depending on a tissue source. For example, canine ADMSCs have higher differentiation potential than MSCs from bone marrow, umbilical cord, amniotic membrane, or placenta (21). In contrast, in horses, chondrogenic (69) and osteogenic potentials (70) seem higher in bone marrow MSCs than in ADMSCs. These results indicate the possibility that MSCs from different animals have different properties indeed. The results of our study confirmed that there is a difference in proliferation potential between species. Canine ADMSCs exhibited higher proliferation potential than feline ADMSCs as cells from dogs had significantly higher (C)CD and significantly shorter CDT than cells from cats.

Similarly, as in proliferation potential, there was a difference in the differentiation potential between canine and feline ADMSCs. While MSCs from both species were able to differentiate into adipocytes, chondrocytes, and osteocytes at early passages, canine cells were able to differentiate also at passage 8, whereas feline cells were able to differentiate at passage 6, but not at later passages. Canine ADMSCs also seemed to possess greater chondrogenic and osteogenic potential than feline cells, as seen after quantification of differentiation images. Adipogenic differentiation was assessed only qualitatively as lipid droplets were very small, and large magnification was required to visualize these droplets. No apparent difference in qualitatively assessed adipogenic differentiation between canine and feline ADMSCs was observed. The small size of lipid droplets formed in canine ADMSCs is in line with the results from other studies, where lipid droplets from differentiated canine bone marrow–derived MSCs were shown to be much smaller than those of human MSCs (50). Also, lipid droplets formed during adipogenesis were reported to be smaller in adipose and bone marrow–derived MSCs than in MSCs from synovium or infrapatellar fat pad (20).

Taken together, lower proliferation and differentiation potential and lower relative cell surface marker expression in feline cells in comparison to canine cells could be explained, at least in part, by the assumption that the feline ADMSC population is more heterogeneous than canine ADMSCs. These findings should be considered in stem cell therapies as population heterogeneity combined with the requirement for the large-scale cell expansion needed for stem cell therapy may significantly impact the *in vitro* characteristics of cells and possibly therapeutic potency of MSCs.

One of the aims of our study was to examine potential sex differences in the viability, proliferation, and differentiation potential in cells from dogs and cats. Sex-related differences have been previously reported in regard to the neurogenic potential (54, 55), immunomodulation (56), and therapeutic efficacy (57) of stem cells in humans and animal models. In our study, the only differences between sexes were observed in osteogenic potential, which was different with cells from dogs and cats at passage 2. This suggests that cells do not differ majorly between sexes in basic parameters such as proliferation and expression of cell surface markers. However, more subtle sex-related differences in animal MSC characteristics might still be present and must not be neglected when studying cell characteristics, especially their therapeutic potentials. Furthermore, sex difference in osteogenic capacity is interesting as it might suggest different properties of cells in regard to sex, and this might reflect in differences in regenerative potential of ADMSCs from different sexes. Therefore, this difference must be further explored in the future study.

In conclusion, our study indicates that animal donor species play an important role with regard to the MSCs' characteristics *in vitro*. ADMSCs from dogs have a higher proliferation rate and better differentiation capacity than cells from cats. Feline cells also seem to be more sensitive to the handling, as viability after 1 day of handling was lower in feline than in canine cells. Furthermore, the percentage of cells expressing stem cell markers was lower in cells derived from cats than in canine cells. However, sex differences were observed only in osteogenic potential in the early passage. Therefore, the results of this study suggest that species should be taken into account when working with MSCs, and protocols for cell isolation, culturing, or therapeutic use cannot be translated from one species to another.

## Data Availability Statement

The raw data supporting the conclusions of this article will be made available by the authors, without undue reservation.

## Ethics Statement

Ethical review and approval was not required for the animal study because all animals were client-owned, and all owners agreed to the collection of tissue and signed informed consent. Since the study was conducted on client-owned animals undergoing a routine clinical procedure with the owner's approval to collect a small piece of adipose tissue, no other approval of the ethical committee was needed according to Slovenian legislation and official opinion from the Administration of the Republic of Slovenia for Food Safety, Veterinary and Plant protection responsible for issuing ethical permits for animal experiments. Written informed consent was obtained from the owners for the participation of their animals in this study.

## Author Contributions

MV performed the experiments. GM planned the experiments and analyzed the data together with MV. MV and GM wrote the manuscript. All authors contributed to the article and approved the submitted version.

## Conflict of Interest

GM is a partial owner of Animacel Ltd. The remaining authors declare that the research was conducted in the absence of any commercial or financial relationships that could be construed as a potential conflict of interest.
